# Editorial: Quorum Network (Sensing/Quenching) in Multidrug-Resistant Pathogens

**DOI:** 10.3389/fcimb.2019.00080

**Published:** 2019-04-03

**Authors:** Rodolfo García-Contreras, Thomas K. Wood, Maria Tomás

**Affiliations:** ^1^Department of Microbiology and Parasitology, Faculty of Medicine, National Autonomous University of Mexico, Mexico City, Mexico; ^2^Department of Chemical Engineering, Pennsylvania State University, University Park, PA, United States; ^3^Microbiology Department, Complejo Hospitalario Universitario A Coruña, Instituto de Investigación Biomédica (INIBIC-CHUAC), Universidad de A Coruña, A Coruña, Spain

**Keywords:** quorum sensing (QS), quorum quenching (QQ), multi-resistant bacteria, enzymes, inhibitors

In relation to the basic aspects of quorum sensing (QS) research, three works were published in this Research Topic. In the first one, Higgins et al. developed a new approach with the *Pseudomona aeruginosa* PAO1-N strain QS regulatory network to study the contribution of the two phenazine-1-carboxylic acid (PCA) operons involved in pyocyanin production. The data of this manuscript show the complexity of the QS cascade in *P. aeruginosa* controlling the production of phenazine secondary metabolites. In the second work, the authors improved the state of the art for surface-enhanced Raman scattering (SERS) spectroscopy for the detection of bioactive extracellular compounds that are involved in interspecies microbial interactions as well as involved in the relationship between the microbes and their hosts (Bodelón et al.); their approach is suitable for *P. aeruginosa* and other multi-resistant pathogens. Highlighting advances in nanotechnology and photonics have increased the possibility of SERS being a more robust analytical tool in the microbiology field. There are several applications of this methodology, including the detection of pathogenic bacteria, and culturing bacterial cells. Moreover, in this review, Bodelón et al. concluded this technology reveals the “hidden” chemistry of microbes which allows the early detection and diagnosis of infectious diseases (Bodelón et al.). Finally, in the third work, Pawar et al. described a theoretical network model using access to a set of small protein interactions (SPINs) together with the whole genome (GPIN) to identify *in silico* proteins involved in the QS in *Proteus mirabilis*. The authors identified new proteins involved in the QS of this pathogen PMI1345, GltB, PMI3678, and RcsC, which could be used as new targets to develop of new treatments for *Proteus mirabilis*.

Current efforts in quorum quenching (QQ) research are dedicated to expanding the existent repertoire of extracts and molecules with anti-virulence properties, useful against important Gram negative multidrug resistant (MDR) bacterial pathogens. Among the newest agents is 1,2-benzenedicarboxylic acid, isolated from an extract of the bacterium *Delftia tsuruhatensis* SJ01, which was obtained from the rhizosphere and is able to inhibit biofilm formation, swarming, and the production of rhamnolipids, pyocyanin, and exoproteases of the reference strain *P. aeruginosa* PAO1 and a clinical isolate, probably by binding and inactivating the LasR receptor (Singh et al.). In addition, bakuchiol isolated from a methanolic seed extract of *Psoralea corylifolia* showed remarkable anti-virulence activity and is also effective for inhibiting the biofilm formation of *P. aeruginosa, Chromobacterium violaceum, Listeria monocytogenes*, and *Serratia marcescens*. Computational analysis demonstrated that this meroterpene binds and disturbs LasR and RhlR structures (Husain et al.) leading to the inhibition of QS-mediated biofilm maturation. Recently, virtual screening 4,687 phytochemicals based docking analysis to detect different potential interactions with the QS receptor of *C. violaceum* the protein CviR. As a consequence, four new inhibitors were identified: sappanol, butein, bavachin, and catechin 7-xyloside. Further, studies using microscale thermophoresis confirmed the predicted interactions, and accordingly, all the compounds were able to able to reduce biofilm formation and the production of violacein (Ravichandran et al.). Also, recently it was discovered that the diterpene alcohol phytol has remarkable anti-QS and anti-virulence properties against *S. marcescens* including *in vitro* inhibition of biofilm formation and swarming motility as well as inhibition of lipase, hemolysin, and exopolysccharide production. In addition, phytol caused an *in vivo* reduction of the bacterial load in kidneys, bladder and urine of Wistar rats inoculated with *S. marcescens* that developed acute pyelonephritis, and the administration of phytol decreased several inflammatory markers and the production of bacterial lipase and protease in the tissues. Since phytol has low toxicity and is widely used in the cosmetic industry, it is possible to envision its application for the treatment of *S. marcescens* in human infections (Srinivasan et al.).

Another way to interfere with QS is the degradation of QS signals like acyl-homoserine lactones (AHL) by acylases and lactonases. This approach has demonstrated better virulence factor attenuation *in vitro* than classical chemical inhibitors, such as the brominated furanone C-30 and 5-fluorouracyl in clinical isolates (Guendouze et al., 2017). Moreover, recently it was demonstrated that such inhibition is more effective under oxidative stress, such as that exerted by the addition of nicotine, since QS is also linked to stress response in *P. aeruginosa* (García-Contreras et al., 2015), particularly in the expression of antioxidant enzymes, such as catalase (Tang et al.). Besides its remarkable effects *in vitro* and in simple infection models, such as in the nematode *C. elegans*, enzymatic QQ by the acylase PvdQ intranasally administrated is also effective in attenuating virulence in murine pulmonary infections caused by *P. aeruginosa*, increasing life expectancy in lethal infections and decreasing damage and inflammation in sub lethal ones (Utari et al.). In addition, lactonases have been used in engineering to reduce biofouling in reverse osmosis systems (Oh et al., 2017); however, this approach is less effective than reducing biofouling by controlling the secondary messenger cyclic diguanylate (Wood et al., 2016). Finally, we highlight new QQ enzymes in isolates of the *Acinetobacter baumannii* (López et al., 2017; Mayer et al.). The first one was described in clinical isolates of *A. baumannii*, the AidA protein (AHL lactonase) described in isolates clinical of the *A. baumannii* (López et al., 2017). This protein showed activity hydrolyzing the 3-oxo-C12-HSL signal which reduced the QS of this bacteria, and also degraded signals from other bacteria. The second QQ enzyme was the A1_2662 protein (AHL lactonase) (Mayer et al.).

Other QS/QQ reference models use *Vibrio* species, which are responsible for severe infections in humans like gastroenteritis, wound infections and septicemia. *Vibrio* spp. is also important pathogens of fish and crustaceans (Liu et al.). The best known is *V. cholerae* due to pandemics. QS in this bacterium is controlled by the global regulator LuxO, which allows the production of virulence factors, such as the cholera toxin and toxin co-regulated pilus formation at low cell densities while downregulating them at high cell densities and promoting detachment from the intestinal epithelial cells via the HapA protease. Due to the central role of LuxO in QS and virulence, it is an adequate target for QS inhibitors. Among the ones discovered so far are 2,3 pyrazine dicarboxylic acid and their derivatives, such as PDCA^py^ that incorporates a pyrrolidine moiety and that is able to downregulate the expression of the toxin and pilus genes and reduce the adhesion of vibrios onto and invasion into (Hema et al.).

Another important *Vibrio* species is *V. campbellii* which is a major problem for aquaculture, since is responsible for “luminescent vibriosis” that causes mass mortality in farmed shrimp. Compound screening based on the inhibition of bioluminisnece, a QS-controlled phenotype, has been useful for the identification of QS inhibitors, and also direct inhibitors of luciferase enzymes, such as SAM461, a 9H-fluroen-9yl vinyl ether derivative that likely binds to the active site of the enzyme and that has no effect on the QS systems. Remarkably, the administration of this compound at low micromolar concentrations protected *Artemia franciscana* against *V. campbellii* infection, suggesting a role of luciferase in virulence and revealing a novel target for anti-virulence therapies (Martín-Rodríguez et al.).

Besides the inhibition of QS receptors and the degradation of the signals, another strategy to interfere with QS and virulence is to inhibit their production by targeting important enzymes participating in their biosynthesis. These are good targets since they only exist in bacteria and are absent in animal hosts. Exploiting this kind of inhibition is in its infancy but already has produced some remarkable results for the inhibition of homoserine lactone synthesis as well as other important signals, such as autoinducer 2, the quinolone PQS and peptide autoinducers of Gram positive bacterial pathogens (Fleitas Martínez et al.).

As another approach, Ma et al. found that 15% of marine bacteria from coral microbial consortia have QQ activity (resulting in the inhibition of biofilm formation and virulence production). A representative of this approach is an isolate of *Staphylococcus hominis* D11 that has genes predicted to be involved in the production of homocysteine thiolactone. The authors purified and analyzed this QS analog and demonstrated that it competes with the auto-inducers producers by *P. aeruginosa*.

Moreover, new approaches in QQ research were described for Gram positive MDR bacterial pathogens like *S. mutans* (Kaur et al.). A new inhibitor of QS, the aromatic 1,3-di-m-tolylurea (DMTU) was identified that acts on the ComDE pathway associated with biofilm formation, and has been studied *in vivo* by Kaur et al. through *S. mutans* infections in Wistar rats. Interestingly, the authors analyzed the incidence of the caries due to the synergistic activity of this QQ compound mixed with fluoride in this animal model. The results show that DMTU decreases the incidence of caries by inhibiting ComA that is an ABC transporter that belongs to the ComDE QS circuit in *S. mutans*. This study also shows that the combination of DMTU with fluoride at lower concentrations can be used as a potential substitute to the current chemotherapeutic approaches to prevent the incidence of dental caries.

Finally, Huedo et al. analyzed in a review article interesting advances in the understanding of the QS/QQ of *S. maltophilia* highlighting those works related with diffusible signal factor (DSF), AHL signaling (acyl homoserine lactones) and the factor Ax21. *S. maltophilia* naturally interacts with the organisms present in its environment. An interesting example of cooperation via DSF is the increment of biofilm formation and antibiotic resistance of *P. aeruginosa* in the lungs. However, in most known cases, *S. maltophilia* inhibits its competitors' QS systems. This is because *S. maltophilia* strains have an extraordinary array of QQ mechanisms including production of virulence factors with quenching activities as well as degradation of AHL and palmitic acid methyl ester activity.

In conclusion, important topics in relation to quorum network (sensing/quenching) of multi-drug resistant pathogens are described in this special issue of Frontiers in Cellular and Infection Microbiology.

## Author Contributions

RG-C and MT wrote the manuscript using papers revised as editors in this Research Topic. TKW participated in the supervision of the writing of the manuscript.

### Conflict of Interest Statement

The authors declare that the research was conducted in the absence of any commercial or financial relationships that could be construed as a potential conflict of interest.
